# Efficacy and safety of outpatient treatment with direct oral anticoagulation in pulmonary embolism

**DOI:** 10.1007/s11239-017-1607-9

**Published:** 2018-01-05

**Authors:** R. Ghazvinian, A. Gottsäter, J. L. Elf

**Affiliations:** Division of Vascular Medicine, Skåne University Hospital, Ruth Lundskogs Gata 10, 205 02 Malmö, Sweden

**Keywords:** Outpatient treatment, Pulmonary embolism, Direct oral anticoagulant, Venous thromboembolism

## Abstract

Anticoagulant treatment of acute pulmonary embolism (PE) has traditionally been hospital-based. The lesser need for monitoring with the increasingly used direct acting oral anticoagulants (DOAC) in comparison to warfarin potentially facilitates outpatient treatment of PE with these drugs. This study aimed to evaluate efficacy and safety of outpatient treatment of PE with DOAC. We extracted data from the Swedish quality registry for patients on oral anticoagulation (AuriculA) for all 245 patients in the southernmost hospital region in Sweden (1.3 million inhabitants) selected for outpatient treatment with of PE with DOAC during 2013–2015. Comorbidites, risk factors, and simplified pulmonary embolism severity index were evaluated at baseline, and death, recurrent venous thromboembolism (VTE), and bleeding was recorded during 6 months of follow-up. Outpatient treatment was defined as discharge from the emergency department within 24 h. During 6 months of follow-up, one patient died during DOAC therapy, the cause of death was unrelated to VTE. No VTE recurrences occured, whereas, one patient experienced major bleeding, and five patients experienced minor bleedings. Outpatient treatment of PE with DOAC is efficient and safe in selected patients.

## Introduction

Venous thromboembolism (VTE), including deep vein thrombosis (DVT) and pulmonary embolism (PE) affects 5% of the population during their lifetime [1]. Patients with VTE are treated with anticoagulant (AC) therapy for a minimun of 3 months to prevent thrombus extension, embolization, and recurrences [1, 2]. Hereafter, the decision to stop or continue treatment depends on the balance between the risks of recurrence (1–10% per year) and bleeding (2–4% per year) [1].

Anticoagulant treatment of acute PE has traditionally been hospital based, with a mean hospital stay of 6 days [3]. Outpatient treatment of PE has been studied already in the 1990s [4] and current international guidelines suggest outpatient treatment for selected low-risk patientes [5], however, the proportion of patients recieving outpatient treatment is still low in most industrialized countries [6]. Different eligibility criteria for outpatient treatment have been used [7–9]. The most well known, the pulmonary embolism severity index (PESI), has been evaluated in a prospective randomised study [10], and the simplified more user-friendly form of PESI (sPESI) compares favorably with the original variant retrospectively [11].

During recent years several direct acting oral anticoagulants (DOAC) with a favorable risk profile have been increasingly used as an alternative to warfarin for VTE treament [12–18]. As the need for monintoring of DOAC treatment is less than for warfarin [18, 19], outpatient treatment of VTE is potentially facilitated by DOAC use. DOAC are nowadays considered as first alternative for DVT in the lower extremities and for in hospital treatment of uncomplicated PE [5], but the safety and efficacy of using DOAC for outpatient treatment of PE has only been evaluated in a small material [20].

VTE patients recruited for randomized controlled trials of AC treatment generally have lower bleeding and recurrence rates than patients in clinical practise, and real life data are therefore valuable for clinical decisions upon AC treatment. In the Swedish quality registry for AC treated patients, AuriculA [21], 25% of the patients currently use DOAC, the indication being VTE in 15–20%. We have previously evaluated results of home-treatment with warfarin in 307 PE patients without noticing unacceptably high complication risks [22]. We now used AuriculA data to clarify risks for VTE recurrence, death, and bleeding during 6 months in 245 consecutively registered patients with acute PE selected for outpatient treatment with DOAC.

## Methods

### Patients

**Fig. 1:**
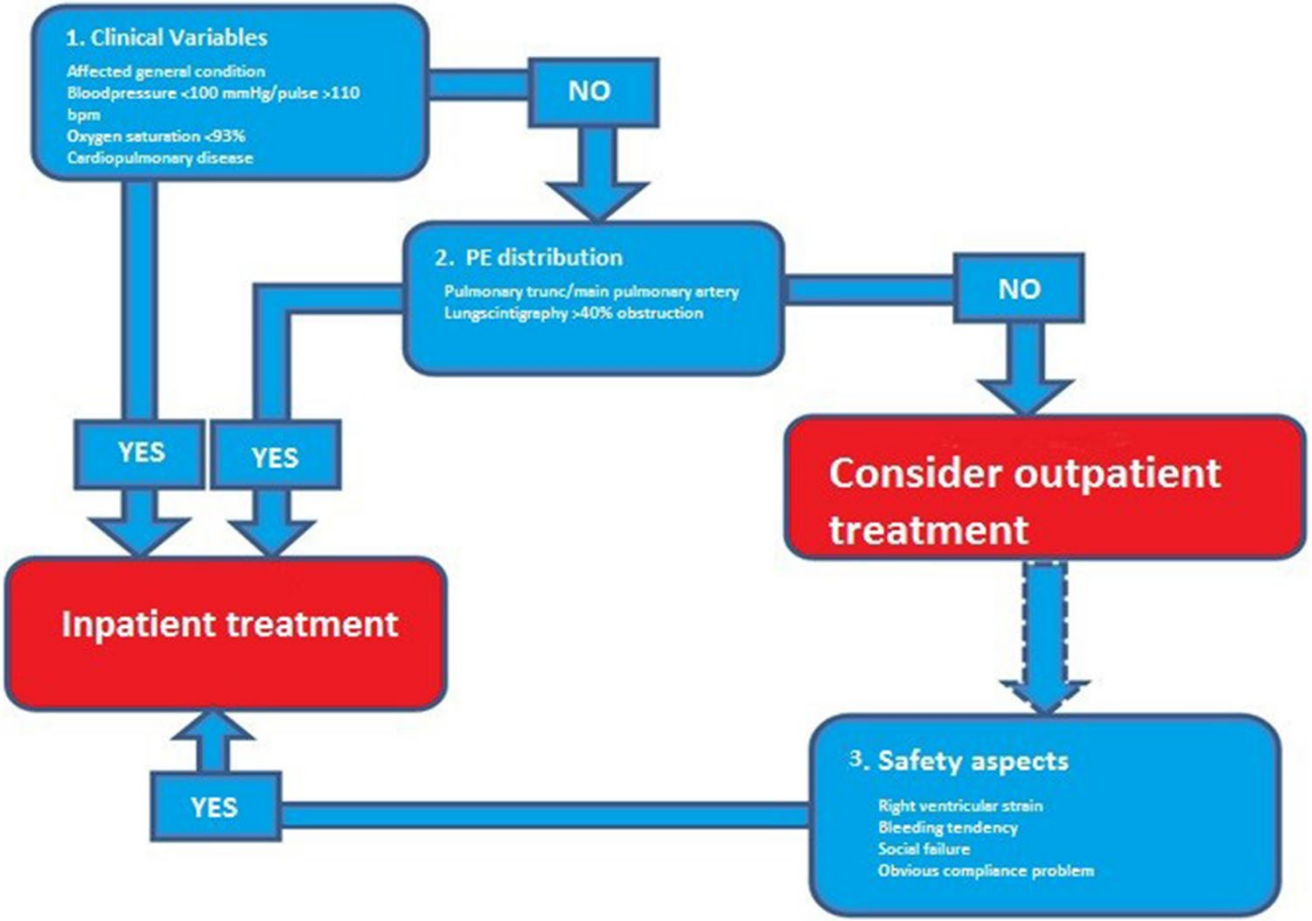
Flow chart used at our institution for selection of patients with PE suitable for outpatient treatment

We extracted data from AuriculA [21] for all 881 patients in the southernmost hospital region in Sweden (1.3 million inhabitants) treated with DOAC for PE (international classification of diseases, tenth-revision [ICD-10] diagnosis codes I26.9 and I26.0) during 2013–2015, a period during which DOAC were gradually replacing warfarin as first line PE treatment. The eight hospitals in the region, out of which one is a tertiary academic hospital, use a flow chart with pragmatic criteria for selection of PE patients suitable for outpatient treatment (Fig. 1). Digital patient files revealed that 245 of the 881 (28%) patients treated with DOAC had been selected for outpatient treatment, i.e., DOAC treatment had been started already during an emergency department (ED) visit not exceeding 24 h.

The following baseline data were retreived from files and imaging databases: symptoms, comorbidities, referral pathway, diagnostic method [computed tomography of the pulmonary arteries (CTPA) or ventilation/perfusion single photon emission computed tomography (V/P SPECT)], sPESI score [11], malignancies diagnosed prior to or at diagnosis of PE, use of central venous catheters (CVC) or oral contraceptives (OC), pregnancy or postpartum state (defined as the first 6 weeks after delivery), and family history defined as VTE in first or second degree relatives, immobilization defined as ≥ 3 days of bedrest, trauma or major surgery, flight travel of > 5 h, cast therapy within the previous month, thrombophilia, ongoing tobacco use, varicose veins or thrombophlebitis, D-dimer [23] (defined as positive if > 0.25 mg/L), troponin T [24] (defined as positive if > 5 ng/L).

Data on mortality, recurrent VTE, and bleeding complications (defined according to the International Society on Thrombosis and Haemostasis [25]) during 6 months after diagnosis had been adjudicated by AuriculA officers before entry into the registry, and files and imaging data for all 881 patients were hereafter reviewed by the authors.

### Statistical analysis

Only descriptive statistics were calculated, using SPSS for Windows, version 23.0 (SPSS Inc, Chicago, IL). Results are expressed as mean ± SD or n (%).

## Results

Baseline characteristics are shown in Table 1. Two of our patients were in week 9 of pregnancy at the time of PE diagnosis, contraindicating DOAC therapy [4, 18, 19]. In one woman, termination of pregnancy was already planned before PE was diagnosed, however, and in the other case this decision was made at the ED visit before initiation of DOAC.

**Table 1:** Characteristics of 245 patients in the Skåne region treated with direct oral anticoagulants (DOAC) because of PE during 2013–2015, n (%) or mean ± SD

Patient characteristics	*n* = 245
Male/female gender	125 (51)/120 (49)
Age (years)	60.0 ± 17.2
Previous DVT	17 (7)
Previous PE	3 (1)
Varicose veins or trombophlebitis	17 (7)
Concomitant diseases	
DVT	3 (1)
Congestive heart failure	27 (11)
COPD	11 (5)
Referral pathway	
From primary care	43 (18)
From hospital	25 (10)
Predisposing factors for VTE	
Pregnancy or post partum^a,b^	2 (2)
Surgical intervention	15 (6)
Cast therapy	8 (3)
Immobilisation	35 (14)
Travel > 5 h	18 (7)
Hormone therapy^a^	23 (9)
Ongoing tobacco use	46 (19)
Family history of VTE	27 (11)
PVC or CVC	2 (1)
Active malignancy	14 (6)
Trauma or fracture	12 (5)
Thrombophilia	29 (12)
Symptoms at admission	
Chest pain	121 (49)
Effort dyspnea	178 (73)
Cough	19 (8)
Leg pain	42 (17)
Other pain	24 (10)
Incidental PE	29 (12)
Investigations	
D-dimer positive^c^	107 (44)
D-dimer NA	89 (36)
TNT positive^c^	110 (45)
TNT NA	57 (23)
CTPA	194 (79)
V/P SPECT	51 (20)
CTPA and V/P SPECT	2 (1)
Echocardiography	48 (20)
Risk stratification, sPESI score	
0	127 (52)
1	98 (40)
2	18 (7)
3	1 (0.4)
4	1 (0.4)

*COPD* Chronic obstructive pulmonary disease, *CTPA* computed tomography of pulmonary arteries, *CVC* central venous catheter, *DVT* deep venous thrombosis, *Echo* echocardiography, *ED* emergency department, *NA* not available, *sPESI* simplified pulmonary embolism severity index, *PVC* peripheral venous catheter, *V*/*P SPECT* ventilation/perfusion single photon emission computed tomography, *VTE* venous thromboembolism

^a^ Percentages of female patients only

^b^ Treatment after decision to terminate pregnancy

^c^ Percentages of patients analyzed

The majority of patients [238 (97%)] were treated for 6 months, whereas DOAC therapy was stopped after 3 months in seven patients. During 6 months of follow-up, one patient died; a 72 years old male patient with cardiac arrest of unknown cause during ongoing treatment with rivaroxaban. Acute echocardiography during rescuscitation showed no dilatation of the right ventricle, but the patient’s relatives declined autopsy.

In total, nine patients underwent objective imaging for suspected recurrent PE during follow up, but no patient was diagnosed with recurrent VTE.

One patient experienced major bleeding during DOAC therapy; a 61 year old male patient was admitted because of haemothorax caused by pneumonia and longlasting cough during DOAC therapy. This caused a reduction of 20 g/L in hemoglobin level, but the patient was hemodynamically stable. He underwent a negative investigation for underlying malignancy, and treatment was changed from DOAC to low molecular weight heparin (LMWH).

Minor bleedings occurred in 5 (2%) patients during follow-up, one patient with repeated epistaxis, one with increased menstrual bleeding, two with macroscopic haematuria, and one with minor gastrointestinal bleeding (Tables 2, 3).

**Table 2:** Treatment data in 245 patients in the Skåne region treated with direct DOAC because of PE during 2013–2015, *n* (%)

	Total
Treatment for 6 months	238 (97)
< 6 months	7 (3)
Dabigatran^a^	2 (1)
Rivaroxaban^a^	225 (92)
Apixaban^a^	23 (9)

^a^ Three patients changed from rivaroxaban to apixaban and one patient from rivaroxaban to dabigatran

**Table 3:** 6 months follow-up of 245 patients in the Skåne region treated with direct DOAC because of PE during 2013–2015, *n* (%)

At 6 months	Total, *n* = 245
Death	1 (0,4)
Major bleeding	1 (0.4)
Minor bleeding	5 (2)
Objective imaging for recurrent PE	9 (4)
Recurrent VTE	0 (0)
Newly detected malignancy	3 (1)

*DVT* Deep venous thromboembolism, *ED* emergency department

During 6 months follow-up, previously unknown malignancies were unveiled in three of the patients with minor bleeding; one gastrointestinal malignancy in an 82 years old male with GI-bleeding, one prostatic and one urinary bladder carcinoma in two male patients, 66 and 73 years old, with macroscopic hematuria.

Change of therapy because of side effects was considered necessary in five; three patients changed from rivaroxaban to apixaban, one from rivaroxaban to dabigatran, and one from rivaroxaban to warfarin.

## Discussion

As DOAC are both effective [4, 12–19] and safe [4, 12–18] for VTE treatment, they are already recommended as first treatment option [4] both in patients treated for DVT in the lower limb and hemodynamically stable patients with PE.

Our retrospective study indicates that DOAC can also be used for outpatient treatment of PE with acceptable efficacy and safety in low risk patients. Current practice of routine hospitalization in this group can therefore be challenged.

The definition of outpatient treatment in our study was the same as in the HESTIA study [7], patients discharged within 24 h after the diagnosis of PE. This definition can be challenged, however. In meta-analysis [8], Zondag identified 13 studies of outpatient treatment of PE with warfarin and LMWH in which the 24 h limit had been applied, and five other studies defining early discharge as within 72 h after admission. None of these studies included patient on DOAC therapy, however. Hereafter, Roy et al. published a systematic review of three meta-analyses and 23 studies [6], involving 3671 patients managed at home (n = 3036) or discharged early (n = 535). Unfortunately, the definition of early discharge was not always clearly defined. The studies reviewed [6] were also heterogenously concerning inclusion criteria and treatment method, and only one study reported on 35 patients treated with DOAC [20].

All studies had at least 3 months of follow-up, with a < 2% overall rate of thromboembolic reccurrences and < 3% of major bleeding. In our study we report no thromboembolic reccurences and only one case (0.4%) of major bleeding.

As the previous studies were almost exclusively performed with warfarin and LMWH, however, our data should not be directly compared to previously published figures. Other important differences between our and previously published materials [6, 8] are patient gender, age, prevalence of concomitant malignancy, and duration of follow-up.

Different patient materials also differ regarding the sets of criteria for selection of PE patients suitable for outpatient treatment. We used a modified [22] version of the sPESI score [11], a validated approach in this respect [26–28]. In previous studies information on PESI or sPESI classification is not always available, and in some cases the HESTIA criteria [7] have been used instead. Apparently both these two methods for patient selection can be used with satisfactory results, and a scientific comparison of the two methods is currently ongoing (clinicaltrials.gov identifier: NCT02811237). Our more pragmatic selection criteria seems to identify more patients suitable for outpatient treatment compared with sPESI since 48% of our study cohort had a non low risk score according to sPESI.

A recent study [29] documented that observation stay, defined as less than 2 days in hospital, was associated with reduced costs compared to conventional inpatient treatment of PE. Furthermore, observation stay was associated with a lower risk for development of hospital-acuqired conditions, whereas death rates, need for thrombolysis, and readmission rate did not differ [29]. Our definition of outpatient treatment as only up to 24 h in hospital might potentially be associated with further savings, and the economic consequences of our strategy will be further evaluated.

The major limitations of the study are its retrospective nature, and the lack of randomization. The selection of patients, both for DOAC instead for warfarin treatment, and for outpatient instead of inpatient treatment of course constitutes another limitation of our study. Our results should therefore not be applied to PE patients in general. According to established criteria for treatment of PE [5], however, a selection process is necessary before patients are recommended outpatient treatment. This fact precludes propensity scoring for comparisons between out- and in-patients with PE. Prospective randomized studies of outpatient treatment of low risk PE patients are indeed warranted.

In conclusion, our 6 month follow-up study showed that outpatient treatment of PE with DOAC is efficient and safe in selected low risk patients with PE.

## Abbreviations

AC: Anticoagulant
CTPA: Computed tomography of the pulmonary arteries
CVC: Central venous catheter
DOAC: Direct acting oral anticoagulant
DVT: Deep vein thrombosis
ED: Emergency department
ICD: International classification of diseases
LMWH: Low molecular weight heparin
OC: Oral contraceptive
PE: Pulmonary embolism
PESI: Pulmonary embolism severity index
sPESI: Simplified pulmonary embolism severity index
VTE: Venous thromboembolism
V/P SPECT: Ventilation/perfusion single photon emission computed tomography
